# The Use of Magnetic Resonance Cholangiopancreatography (MRCP) in the Setting of Acute Pancreatitis: When is it Most Useful?

**DOI:** 10.51894/001c.5963

**Published:** 2017-08-24

**Authors:** Karlin Sevensma, Miranda Allen, Rebecca Harden, William Corser

**Affiliations:** 1 Metro Health General Surgery Program Director, Wyoming, MI; 2 Metro Health General Surgery Residency Program, PGY 2, Wyoming, MI; 3 Statewide Campus System, MSU College of Osteopathic Medicine, East Lansing, MI

**Keywords:** elevated lft, cholangiopancreatography, cholangiography, acute pancreatitis

## Abstract

**CONTEXT:**

The usefulness of MRCP in the workup of acute pancreatitis has long been debated.

**METHODS:**

2013-2016 chart review data were collected by the authors from adult patients with acute pancreatitis who also had received Magnetic Resonance Cholangiopancreatography (MRCP). Those patients were categorized by diagnosis and according to whether or not the MRCP changed healthcare services.

**RESULTS:**

Changes in care were significantly correlated with diagnosis and elevated liver function tests (LFT). The patients who benefitted most from MRCP were those with acute gallstone pancreatitis (r = 0.298, n = 109, p = 0.002) and patients with elevated LFT (r = 0.219, n = 89, p= 0.040). The most common way that MRCP influenced the care of patients with acute gallstone pancreatitis was by allowing providers to forego intraoperative cholangiogram (IOC) when MRCP results were negative (r = 0.335, n = 109, p < 0.001).

**CONCLUSIONS:**

The authors conclude that this was not the most cost effective management practice since the cost of intraoperative cholangiogram was about one quarter that of MRCP. Limiting MRCP use in patients with acute gallstone pancreatitis and preferentially using IOC at the time of surgery can likely decrease hospital costs without compromising care.

## INTRODUCTION

Magnetic resonance cholangiopancreatography (MRCP) is an expensive study, averaging over $2,000.^1^ Although MRCP has long been thought to be a reliable predictor of choledocholithiasis, patient selection criteria for MRCP could potentially be refined to improve its cost-effective use. During the past 10 years, MRCP has been increasingly utilized in the evaluation of bile duct pathology, more frequently shifting the care of patients with acute gallstone pancreatitis to pre-operative biliary duct evaluation.^1^ As intraoperative cholangiogram (IOC) and endoscopic retrograde cholangiopancreatography (ERCP) are now less often ordered, the management of common bile duct stones has been found to be more often affected by the availability of instrumentation, personnel and skills rather than cost-effectiveness.^2^

Numerous studies have shown MRCP to be nearly as accurate as ERCP for detecting common bile duct pathology ^2–4^ In the authors’ institution, MRCP is one of the first studies performed on patients admitted with presumed choledocholithiasis or acute gallstone pancreatitis. However, recent studies have called into question the routine use of MRCP in these patients, especially given that many patients with acute gallstone pancreatitis do not have choledocholithiasis at time of presentation. ^5^ Cavdar et al. demonstrated that delayed MRCP at seven days after the onset of symptoms was more accurate than on admission.^5^ This study group also recommended that MRCP in acute biliary pancreatitis should be withheld for at least a week if there is no clinical worsening since many patients with choledocholithiasis will spontaneously pass their stones.^5^ Lin et al. determined that laparoscopic cholecystectomy and IOC were associated with a shorter length of stay than preoperative MRCP or ERCP in patients with suspected choledocholithiasis.^6^

Richard et al. found that MRCP had a high rate of false normal results (Negative Predictive Validity 77%) compared with IOC and is not as accurate as more invasive diagnostic techniques. ^7^ This group concluded that there was no need for pre-operative MRCP in patients with suspected choledocholithiasis, and that cholecystectomy with IOC was preferred.^7^ Although one study also concluded that liver function tests (LFT) are not predictive of CBD pathology on MRCP, ^8^ the results from other studies have shown them to be somewhat predictive. ^9,10^

### Purpose of Study

This study was conducted to examine the use of MRCP and its effectiveness in altering care in a sample of hospital patients with different types of acute pancreatitis, specifically gallstone and acute pancreatitis from other causes, such as alcohol, hyperlipidemia, drug-induced and idiopathic. This study also examined whether LFT on admission or hospital Day One were predictive of MRCP revealing biliary duct pathology. Before the study, the authors hypothesized that MRCP was potentially being over-utilized and might offer limited benefit to only certain types of patients. The authors anticipated that this could save hospital costs if those patients could be identified and workup re-directed.

## METHODS

This study was reviewed and approved by the Metro Health Hospital Institutional Review Board. A waiver of informed consent was received. Following this approval, retrospective chart reviews were completed by the first three authors for all adults admitted to the hospital between January 2013 and May 2016 with a diagnosis of “acute pancreatitis” at any time during their hospital stays. Information regarding their admitting provider, date of admission, date of discharge, date of MRCP, findings of MRCP, interpreting radiologist, liver function tests, ultrasound findings, date of laparoscopic cholecystectomy, intraoperative cholangiogram and findings, date of ERCP and findings, and type of acute pancreatitis were also collected.

The following inclusion and exclusion criteria were observed:

Inclusion Criteria:

- Patients 18 years of age and older
- Admitted to the hospital and diagnosed with acute pancreatitis between January of 2013 and May of 2016
- Underwent MRCP for evaluation

Exclusion Criteria:

- Patients under 18 years of age
- Diagnosis of chronic pancreatitis

MRCP results were then classified as “positive” if there was visible pathology causing bile duct obstruction (e.g., stone, sludge, mass or stricture.) For other cases in which there was no visible cause of duct obstruction, results were classified as “negative.” Bilirubin and transaminases LFT values were categorized as “normal” or “elevated” for analyses based upon standard normal ranges for the hospital laboratory. Finally, whether each sample patients’ care had changed based upon MRCP result was determined. A change in care was defined as follows. For patients with acute gallstone pancreatitis, a change in care was considered to have occurred if the patient had preoperative ERCP, an ERCP ordered instead of surgery or if no IOC was performed at the time of surgery since the MRCP was negative. For patients with other types of acute pancreatitis, an MRCP was considered to have changed care if an ERCP was ordered as a result of the MRCP results.

### Statistical Analysis

After data cleaning, a series of descriptive statistics (frequencies, cross-tabulation charts) Pearson product-moment bivariate correlational procedures were first completed to examine for both missing data patterns and study data distributional patterns of key variables. A basic two-tailed stepwise binary logistic regression predictive modeling procedure was then completed ^11^ to examine for statistically significant influences on whether sample MRCP patients’ care was changed as a result of having received an MRCP. A coefficient Alpha two-tailed significance p value level of 0.05 was observed. All analyses were completed using S.P.S.S. version 22 analytic software. ^12^ Statistical analysis was performed by the fourth author.

## RESULTS

Four hundred and twelve patient encounters for acute pancreatitis were initially identified for analysis. A total analytic sample of 109 patients (26.5% of total population) from the initial data set had received MRCP as part of their workup for acute pancreatitis. This subgroup of patients was evaluated for: a) correlation of the MRCP result with elevation in transaminases and bilirubin LFT values, both on admission and hospital Day One, and b) how MRCP results may have changed their patient care.

Of the 109 patients undergoing an MRCP, 52 (47.7% of analytic sample) were found to have acute gallstone pancreatitis, with 57 (52.2%) diagnosed with acute pancreatitis of another type. Of the patients with acute gallstone pancreatitis, four (7.7%) patients had received a pre-operative ERCP as a result of the MRCP being positive for stone or sludge. Twenty-five (48.1%) of acute gallstone pancreatitis patients had received no IOC at the time of surgery because their MRCP was negative. Five (9.6%) patients with acute gallstone pancreatitis had an ERCP ordered to evaluate duct abnormalities and did not have surgery. Two (4.0%) patients did not fit into any of the above groups and were categorized as “Other.” Sixteen (30.7%) patients with gallstone pancreatitis had no change in their care due to their MRCP results.

This finding indicates that the patient either did not have surgery during admission for gallstones (six patients) or had undergone an IOC during surgery despite a negative MRCP (ten patients). In one of those cases, the IOC was positive for a filling defect that cleared with glucagon. In those patients with non-gallstone acute pancreatitis, 52 (91.2% of analytic sample) patients did not have any change made in their care as their MRCPs were negative. An ERCP was ordered in the remaining five (8.8%) patients, constituting a change in care. (See Table 1)

**Table 1:** Distribution of Patient Subgroups and Change in Care (N = 109 patients)

	Procedures ordered or not ordered based on MRCP result	N (% of category)
Gallstone pancreatitisN=52(47.8% of total sample)		
	ERCP pre-op	4 (7.7%)
	No IOC	25 (48.1%)
	ERCP ordered	5 (9.6%)
	other	2 (4.0%)
	No change in care	16 (30.7%)
Non-gallstone pancreatitisN=57(52.2% of total sample*)*		
	ERCP	5 (8.8%)
	No change in care	52 (91.2%)
		**TOTAL 109**

**Figure 1:**
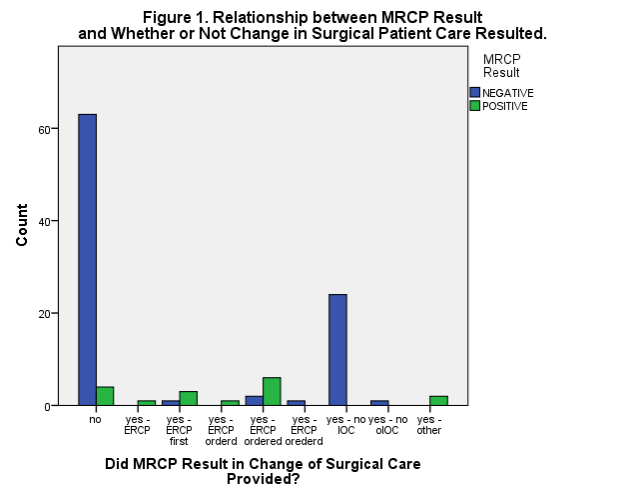
Relationship between MRCP Result and Potential Changes in Patient Care

**Figure 2:**
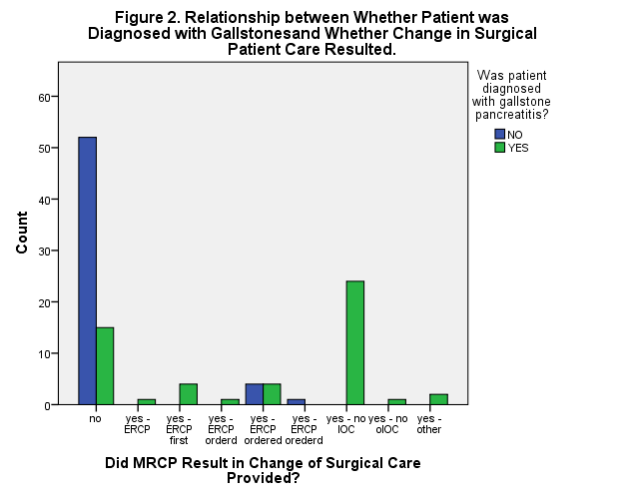
Relationship Between Whether Patient was Diagnosed with Gallstones and Whether Change in Patient Care Resulted

Overall, these patients’ MRCP results (i.e., negative or positive) were significantly correlated with: a) whether or not at least one LFT result was elevated at admission and/or Day One (r = 0.219, n = 89, p= 0.040), and b) whether or not a patient was diagnosed with acute gallstone pancreatitis (r = 0.298, n = 109, p = 0.002) (Figures 1 & 2). The documented MRCP results (i.e., either negative or positive) were also significantly correlated with whether or not the surgical care provided to that patient was altered (r = 0.335, n = 109, p < 0.001).

Finally, a stepwise binary logistic regression model, in which potential predictive terms are entered into the preliminary predictive model and retained or removed (if initial Alpha significance level was greater than 0.010) was completed. This type of predictive model is not based on any assumption of normal distributional patterns and requires a dichotomous (i.e. *Yes/No*) outcome. In the final predictive model, two terms “survived” as significant influences concerning whether sample patient’s care was adjusted: a) whether or not the patient had been diagnosed with acute gallstone pancreatitis (Wald chi square = 3.888, p < 0.001 with df = 1), and b) whether or not patient had at least one elevated LFT result during the first 48 hours of their hospital stay (Wald chi square = 3.888, p = 0.049, with df = 1).

In our institution, an MRCP costs $2,014 (USD). The cost of an IOC in this setting is somewhat less straightforward, varying on the amount of operative time used for IOC. If an additional operative time of 15 minutes is used for the calculation, an IOC at our institution costs $748 (USD), nearly a third the cost of MRCP. (See Table 2 for IOC cost calculation.)

**Table 2:** Calculation of Total Intraoperative Cholangiogram (IOC) Costs

Component of cost	Cost
Surgeon Reimbursement(Medicare cost)	$58
Radiologist Interpretation(Medicare)	$19
Anesthesiologist Charges15 minutes(Medicare)	$22
Hospital Operating Room charges(15 minutes)	$263
Fluoroscopy Charges(C-arm, contrast, technologist)	$264
Supplies for IOC(catheter, syringes, connectors)	$122
Total	**$748 (USD)**

## DISCUSSION

Based on these results, the authors’ overall hypothesis that MRCP would offer limited benefit to certain types of acute pancreatitis patients was supported. The patients who showed the most benefit from having received an MRCP were those with acute gallstone pancreatitis and those with elevated LFT.

Since MRCP is a relatively expensive study, it should be reserved for patients who will more likely benefit from it. These study results indicate that patients with acute gallstone pancreatitis benefited more from MRCP than patients with other causes of acute pancreatitis. Unfortunately, much of this benefit was in the form of avoiding an IOC. The problem with using MRCP in this way is that the test has been demonstrated to have a high false negative rate in some studies. ^7,13^

MRCP has not been shown to be as cost-effective as IOC for evaluating the duct for pathology, nor has it been shown to be as accurate.^6,9^ ^7,14^ Pre-operative MRCP is more expensive than IOC and may also prolong hospital length of stay.^7^ In our study, using IOC preferentially to MRCP in patients with acute gallstone pancreatitis undergoing laparoscopic cholecystectomy would have decreased the need for an MRCP by about 25% of all cases. However, if a patient is not considered to be a candidate for laparoscopic cholecystectomy (e.g., in patients with other etiologies of acute pancreatitis), this approach is not as viable.^6,7^ When possible, IOC should be done preferentially to MRCP for the purposes of accuracy, cost-effectiveness, and efficiency. Based on these study findings, MRCP should not be ordered as a first line test in patients with acute pancreatitis not attributable to gallstones since it is unlikely to change care or affect management decisions.

As indicated in our data, patients with elevated transaminases and/or bilirubin LFT within 48 hours of admission were more likely to benefit from MRCP than those who did not have elevated lab values. This suggests that MRCP should generally be reserved for patients with elevated LFT and only ordered with reservation when LFT are normal.

This study’s scope was limited secondary to being a single-site, retrospective study. The conclusions drawn from this study should be further evaluated in larger multi-site prospective studies examining preferential use of IOC over MRCP in patients with acute gallstone pancreatitis. Furthermore, many of the acute pancreatitis patients in this sample from causes other than gallstones did not undergo MRCP, perhaps obscuring the potential measured benefits of MRCP for those patients. Due to this smaller-sized convenience sample, the authors may have also lacked an adequate level of statistical power to detect other meaningful sample subgroup differences.

In this sample, MRCP was most useful in patients with acute gallstone pancreatitis, with the largest group of patients who benefited from MRCP being patients who had not undergone IOC at the time of laparoscopic cholecystectomy due to negative MRCP results. Due to the false negative rate of MRCP, using this test in this way is problematic. Since the majority of sample patients with gallstone pancreatitis did not have choledocholithiasis at the time of imaging or surgery, IOC may have been a more cost-effective way to rule out choledocholithiasis than MRCP. IOC can also be converted to a therapeutic procedure if the surgeon is trained in laparoscopic duct exploration and the necessary equipment is available. Selectively ordering an MRCP in patients with elevated LFT and performing an IOC during surgery in place of preoperative MRCP for acute gallstone pancreatitis patients will tend to decrease healthcare costs without compromising patient care.

### Conflict of Interest

The authors declare no conflict of interest.
